# Dose Book and Manual of Prescription Writing

**Published:** 1895-04

**Authors:** 


DOSE BOOK AND MANUAL OF PRESCRIPTION WRITING—With a list of the official drugs and preparations, and also many of the newer remedies now frequently used, with their doses. By E. Q. Thornton, M. D., Ph. G., Demonstrator of Therapeutics, Jefferson Medical College of Philadelphia; Acting Assistant Surgeon United States Marine Hospital Service. Price, $1.25. Philadelphia: W. B. Saunders. 1895.

We venture to assert that no branch is so little taught in medical schools as prescription writing and dosage, and that the average graduate finds his greatest difficulty in writing correct prescriptions. While there should be no need of books on this subject, the schools are responsible for such a demand, and Professor Thornton has supplied us with a very valuable and complete book.

Part first is devoted to weights and measures, with the method of converting metric weights, measures and lengths into those more ordinarily used.

Part second goes fully into prescription writing and deals principally with "Grammatical Construction of Prescriptions;" "Principles of Medical Combinations;" "Incompatibilities;" "Therapeutic Incompatibility," etc.

Part five gives dosage, methods and frequency of administration; inunctions; fumigations; vapor baths, with age, temperament, idiosyncrasies, climate, habit, etc.

Dr. Thornton has certainly taken great pains in the preparation of this scholarly work, which will make it a choice addition to any library, and we heartily recommend it to those who find difficulty in writing correct prescriptions.

